# Determining the effects of nitrogen rate on cotton root growth and distribution with soil cores and minirhizotrons

**DOI:** 10.1371/journal.pone.0197284

**Published:** 2018-05-11

**Authors:** Jing Chen, Liantao Liu, Zhanbiao Wang, Hongchun Sun, Yongjiang Zhang, Zhanyuan Lu, Cundong Li

**Affiliations:** 1 Department of Agronomy, Agricultural University of Hebei / State Key Laboratory of Cotton Biology (Hebei Base) - Laboratory of Crop Growth Regulation, Baoding, Hebei Province, China; 2 Institute of Cotton Research of Chinese Academy of Agricultural Sciences, Anyang, Henan Province, China; 3 Inner Mongolia Academy of Agricultural & Animal Husbandry Sciences, Huhhot, Inner Mongolia, China; Hainan University, CHINA

## Abstract

Cotton root growth can be affected by different nitrogen fertilizer rates. The objective of the present study was to quantify the effects of nitrogen fertilization rate on cotton root growth and distribution using minirhizotron and soil coring methods. A secondary objective was to evaluate the minirhizotron method as a tool for determining nitrogen application rates using the root distribution as an index. This study was conducted on a Bt cotton cultivar (Jimian 958) under four nitrogen fertilization rates, i.e., 0, 120, 240 and 480 kg ha^-1^ (control, low, moderate and high levels, respectively), in the Yellow River basin of China from 2013–2015. The sampling process, details of each method as well as the root morphology and root distribution were measured. The operational processes, time and labor needed for the soil core method were all greater than those for the minirhizotron method. The total root length density and the length density in most soil layers, especially in the upper soil layers, first increased but then decreased as nitrogen fertilization increased, and the same trend was observed for both methods. Compared with N0, the total root length density under moderate nitrogen fertilization by the soil coring method increased by more than 94.82%, in 2014 and 61.11% in 2015; while by the minirhizotron method the corresponding values were 28.24% in 2014 and 57.47%, in 2015. Most roots were distributed in the shallow soil layers (0–60 cm) in each method. However, the root distribution with the soil coring method (>73.11%) was greater than that with the minirhizotron method (>47.07%). The correlations between the root morphology indexes of shallow soil depth measured using the two methods were generally significant, with correlative coefficients greater than 0.334. We concluded that the minirhizotron method could be used for cotton root analysis and most cotton roots distributed in upper soil layers (0-60cm). In addition, a moderate nitrogen rate (240 kg ha^-1^) could increase root growth, especially in the shallow soil layers. The differences observed with the minirhizotron method were clearer than those observed with the soil coring method.

## Introduction

Roots play an important role in plant growth, as they are in direct contact with the soil and are the main organs that absorb water and nutrition [1]. In addition, roots enable crop synthesis and the transportation of physiological activators, such as cytokinin (CTK) and auxin (IAA) [2–4]. Furthermore, root growth and distribution are closely correlated with crop development and yield [5]. Therefore, the root system is very important in crop production, however, root systems have been frequently overlooked [6]. Root growth is affected by a number of factors, among which nitrogen is considered a key one [7–9]. The roots of cotton are sensitive to nitrogen stress. Cotton root morphology, root biomass are affected by nitrogen fertilization [7]. Adequate nitrogen can improve the root length density and root activity in the 40–120 cm soil layer under mulch drip irrigation [10, 11]. Nitrogen fertilizer reduces root length, root surface area, volume, and diameter under drip irrigation with saline water [12]. Fertilizer affects root distribution, cotton root length density, root volume density in the 0–40 cm soil layer is decreased by nitrogen application [13]. In addition, nitrogen application can increase cotton root nitrate reductase activity and protective enzyme [14–16]. Research on the effects of nitrogen fertilization on the roots morphology and distribution of cotton under field conditions is of great importance.

Several methods are used to study roots, and each has its merits and faults [17]. Destructive sampling has been routinely used in crop root research [18–19]. Intensive labor is needed to dig out soil block and separate living roots from the soil [20]. In addition, the accuracy of root density measurements can be affected by the spatial variability of root distribution. The minirhizotron method provides an in situ, nondestructive method for observing root growth using sequential photographs [21]. This method involves a transparent soil tube, scanner, collapsible measuring rod and computer. The use of the minirhizotron method averts one of the main constraints of root studies, namely, the time and labor needed for sampling and selecting roots for further quantification. Furthermore, the minirhizotron method allows dynamic studies because each individual root can be tracked over time, which makes it possible to simultaneously investigate root production and mortality [22], fine root turnover [23] and irrigation water management [24]. Another advantage of the minirhizotron method is that images scanned at different depths clearly reflect the root growth and distribution throughout the soil profile. However, this method has certain drawbacks, such as increased root concentration near the soil tube interfaces, statistical problems, and the high cost and difficulty of installing tubes [25]; in addition, minirhizotron tube material can affect roots, which was noted by Jennifer et al. [26]. The minirhizotron method has been used in many studies. The roots within the surface soil are affected by the minirhizotron method [27]. Pierret concluds that fine roots are the major component of root systems of most annual and perennial plants using minirhizotron method [28]. Hulugalle suggests that most of the fine root of cotton occurred in the surface 0.5m, with low density in the subsoil under waterlogged conditions [29]. Bland [30] and Merrill [31] find a theoretical model for the conversion of minirhizotron data of cotton roots to root length density. The quantitative estimates of root length density observed using the minirhizotron and other methods are not the same [32]. Therefore, the minirhizotron method still needs to be calibrated against various traditional methods for increase the utility of the method.

The objective of the present study was to quantify the effects of nitrogen fertilization rates on cotton root growth and distribution using the minirhizotron and soil coring methods under field conditions. A secondary objective was to evaluate the minirhizotron method as a tool for determining nitrogen application rates using the root distribution as an index.

## Materials and methods

### Experimental site and cultivar

**Fig 1:**
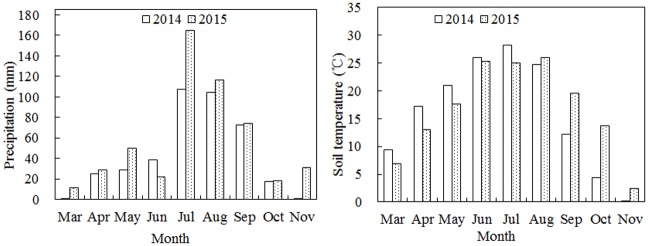
Monthly precipitation distribution and average soil temperature during the cotton growth period in 2014 and 2015 in Baoding (China).

The experiment was conducted at the experimental station of Hebei Agricultural University in Baoding district (38.85°N, 115.30°E), Hebei Province, in the Yellow River basin of China, in 2014 and 2015. The experimental site has a temperate climate. The rainfall and temperatures during the 2014 and 2015 are given in Fig 1. The total precipitation during the growing seasons was 396.0 mm in 2014 and 517.9 mm in 2015. The soil was a loam with an organic matter concentration of 16.4 g kg^-1^, a total nitrogen concentration of 1.13 g kg^-1^, an available phosphorus concentration of 15.36 mg kg^-1^ and available potassium concentration of 191 mg kg^-1^; the fertility level was moderate. The soil bulk density was 1.31, and the pH was 5.9. The experiment was conducted in the same field for two years to avoid soil differences. Acid-delinted seeds were treated with fludioxonil (Shileshi, Syngenta Crop Protection Company, Switzerland).

### Experimental design

A completely randomized block design consisting of three replications was used for the study. The treatments included nitrogen fertilizer (in the form of urea) at rates of 0, 120, 240, and 480 kg N ha^-1^, hereafter referred to as the control, low, moderate and high nitrogen treatments, respectively. A nitrogen rate of 240 kg ha^-1^ was the typical fertilization rate used locally. Each plot contained fourteen rows of cotton and constituted an area of 115 m^2^.

Half of each nitrogen rate was applied basally before sowing, and the remaining half was furrow dressed at the flowering stage and peak boll-setting stage. The amounts of P_2_O_5_ and K_2_O were 135 kg ha^-1^ and 75 kg ha^-1^, respectively, and both were applied basally. Urea (N, 46%) was used as a nitrogen fertilizer, calcium superphosphate (P_2_O_5_, 12%) was used as a phosphate fertilizer, and potassium chloride (K_2_O, 60%) was used as a potassium fertilizer.

### Field management

The field was irrigated (1100 m^3^ ha^-1^) 10–15 days before sowing each year. The soil was then rotary plowed (20 cm) and harrowed when the soil moisture was considered acceptable. The cotton was sown on 24 April 2014 and on 22 April 2015.

The planting density was 45,000 plants ha^-1^, and the row distances were 0.50 and 1.00 m. Using the mechanical sowing method, four or five seeds were dropped per hill. The seeds were then quickly covered by moist soil. A plastic film (0.008 mm) was mulched along the row after sowing to reduce evaporation, increase soil temperature and control weeds.

In accordance with local practices, holes were poked in the mulching film to allow the seedlings emerge. At the two-leaf stage, one vigorous plant was then left per hill; the seedlings were thinned to 45,000 plants ha^-1^. Near the squaring stage, the vegetative branches at the bottom of the cotton main stem were removed (11 June 2014 and 14 June 2015). During the flowering and boll period, the terminal buds at the top of the main stem were also removed (20 July in 2014 and 21 July 2015). Also at the flowering and boll period, all the plots were irrigated (late June of 2014 and 2015). Pest and weed control and other cultivation practices were performed in accordance with cultivation practices.

### Sampling and investigation

#### Root sampling

The growth characteristics of cotton roots in this experiment were measured using the minirhizotron method and soil coring method. The roots were sampled four times, namely, at the budding, early flowering, flowering and boll period stages, as well as the boll opening period.

#### Soil coring method

Root samples were obtained by digging out soil blocks in the flowering and boll period (on 14 August 2014 and 19 August 2015). A cylindrical soil core with a 70 mm inner diameter was used to dig out the roots. Three plants were selected per plot. Three samples were taken at the distance of 25 cm from each plant. The nine samples were treated separately.

Soil cores were obtained by soil coring (70 mm inner diameter). Eight soil samples to a depth of 150 cm were successively removed, and each soil core was sampled for eight soil blocks at different depths (0–15, 15–30, 30–45, 45–60, 60–75, 75–90, 90–120 and 120–150 cm). After the soil blocks were obtained, the soil blocks of each sample were placed in separate marked plastic bags. The soil blocks were then placed into mesh bags (0.15 mm diameter), after which the bags were placed in water containing an ultrasonic transducer (the ultrasonic waves help disperse the clay aggregates) and shaken for 10 minutes. The water washing removed the soil particles, leaving behind the roots and some large debris in the mesh bag. The roots and large debris were subsequently brought to the laboratory. They were placed in a transparent box, and white paper was put beneath the box to increase the visibility of the roots. The living cotton roots were collected with tweezers by the same experienced operators each year.

The roots were scanned using EPSON-V700 scanner. For scanning, the roots were placed in the transparent polyvinyl chloride (PVC) box, and water was added to a depth of 2mm. The roots were placed separately to reduce error. The storage format of picture is “tiff”. The root length, root projected area, root surface area and root volume were determined using WinRHIZO 7.6.1 software. The density of root length, root projected area, root surface area and root volume were calculated by applying the following formulas: root length density (RLD) = L/v, root projected area density (RPAD) = PA/v, root surface area density (RSAD) = SA/v, and root volume density (RVD) = V/v. L, PA, SA, and V represent the root length, root projected area, root surface area and root volume, respectively, as measured by the WinRHIZO software; v is the volume of each soil sample.

#### Minirhizotron method

Eighteen minirhizotron tubes per treatment were installed at an angle of 45° to vertical, parallel to the plant row, and at a distance of 25 cm (halfway between rows) from the cotton plants. The tubes were made of plastic, and the bottom was sealed. The total length of each tube was 200 cm, and the tubes reached a total depth of 150 cm (with 15–20 cm left outside the soil). Light was restricted from the aboveground section of each tube by a black cover. The minirhizotron tubes were installed during the winter of 2013 to ensure that the soil would be well distributed around the tubes and prevent roots from growing around the tubes.

To measure root growth characteristics, images were recorded by the scanner (Manufacturer: CID Bio-Science, Inc. 1554 NE 3^rd^ Ave Camas, WA 98607; Models: Model CI-600). The scanner was connected to a laptop computer and was inserted into each tube during measurements. Images were captured at 9 positions along the tube with the aid of a connecting rod and separated by intervals of 20 cm.

The images were saved as “bmp” format and then analyzed with WinRHIZO software, which provided values of root length, root projected area, root surface area, and root volume by tracings of the boundaries of each root using a mouse.

The root length, root projected area, root surface area, and root volume per unit soil volume were obtained by applying the following formulas: root length density (RLD) = L/(A×DOF), root projected area density (RPAD) = PA/(A×DOF), root surface area density (RSAD) = SA/(A×DOF), and root volume density (RVD) = V/(A×DOF). L, PA, SA, and V represent the root length, root projected area, root surface area and root volume, respectively, as observed by the WinRHIZO software. A represented the area of each image, and DOF was the thickness of the soil of the root being observed, which was 2 mm in this study.

### Statistical analysis

Analysis of variance (ANOVA) was performed with a complete randomized analysis using SPSS version 17.0. Differences between treatments were analyzed using Duncan’s test of one factor ANOVA at the 5% probability level, with nitrogen as the main effect. Correlation analyses were conducted using Spearman’s method of SPSS version 17.0.

## Results

### Sampling procedures of the soil coring method and minirhizotron method

First, the number of processes were different: the soil coring method involved six processes, including digging out the soil blocks, bagging the soil blocks, washing the soil blocks, picking out the roots, scanning the roots and analyzing the root images; the minirhizotron method involved only two processes, namely, root scanning and root image analysis. Second, the sampling volume of the soil coring method was greater than minirhizotron method; the volumes were 467.59 cm^3^ and 79.17 cm^3^, respectively (Table 1).

**Table 1:** Comparison of sampling procedures using the soil coring method and minirhizotron method.

Sampling method	Number of processes	Sampling volume (cm^3^)	Sampling time (min)	Data analysis time (min)	Number of Labors
Soil coring method	6	467.59	10–20	10–80	3
Minirhizotron method	2	79.17	2–3	5–30	2

In addition, sampling required more time in the soil coring method: one sample generally took approximately 10 minutes, and more time was required when the soil layer was of a heavy texture—upwards of 20–30 minutes; using the minirhizotron method, each sampling required only 2–3 minutes. Compared with the minirhizotron method, the soil coring method required more time to analyze the data. The time needed for each sample ranged from 10 to 80 minutes; In minirhizotron method, all the roots are marked on a computer with a mouse. The time needed ranged from 5 to 30 minutes.

In addition, at least three people were needed to perform the complex and difficult processes involved in the soil coring method, while two people were needed in minirhizotron method. The results showed that the minirhizotron method was simpler and more convenient than soil coring method.

### Total cotton root length density as measured by the soil coring method and minirhizotron method under different nitrogen treatments

**Fig 2:**
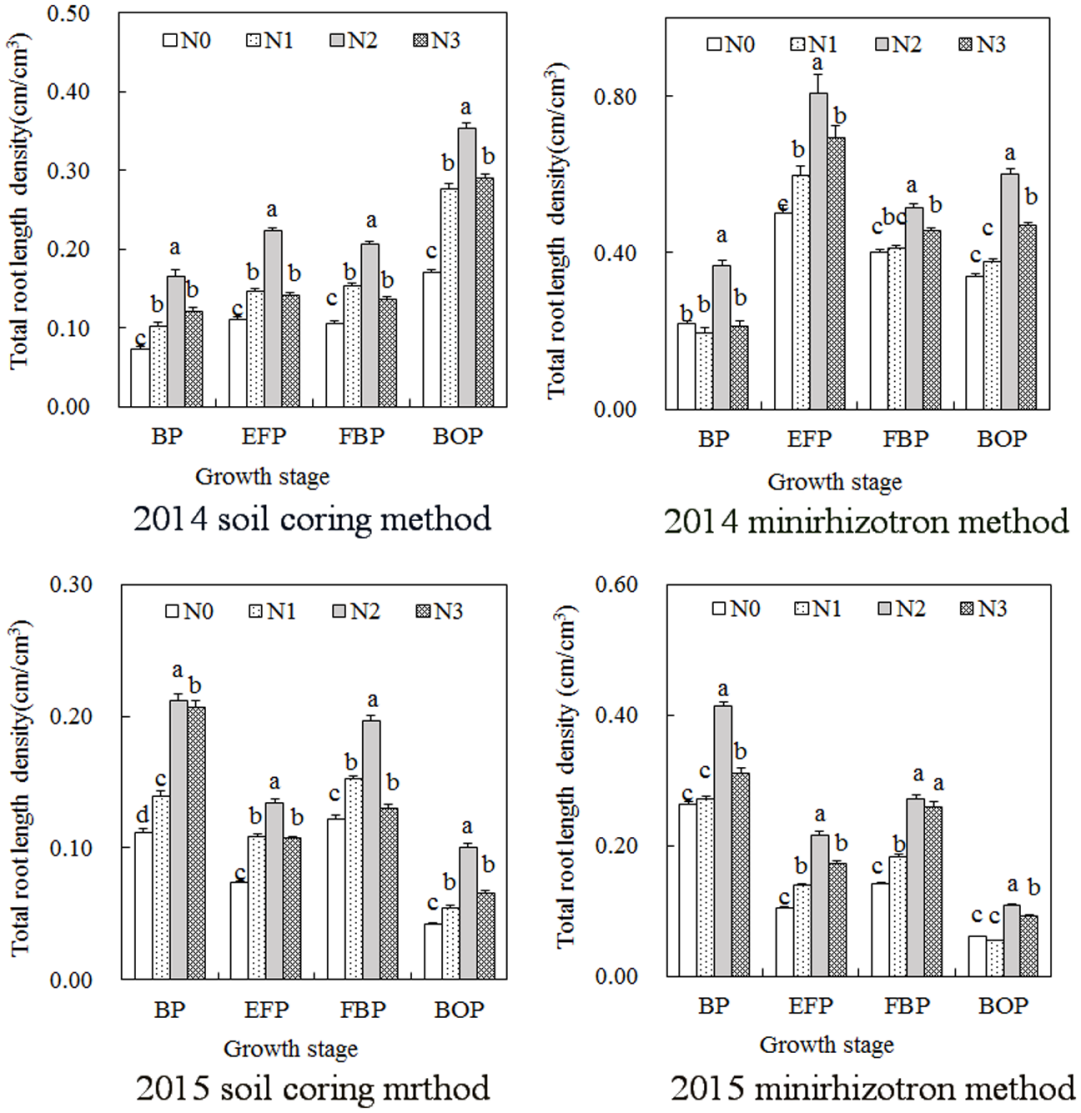
Total root length density of cotton under different nitrogen treatments using the soil coring method and minirhizotron method.

The total root length density measured using the soil coring method was less than that measured using the minirhizotron method (Fig 2); the results were consistent in both years. Nitrogen fertilization increased the total root length density. The moderate nitrogen fertilization resulted in the greatest total root length density, which was significantly greater than that resulting from N0, and the same results were obtained in both methods. Compared with that under N0, the total root length density under moderate nitrogen fertilization by the soil coring method at the budding stage, early flowering stage, flowering and boll opening stage, and boll opening period increased by 124.69%, 101.58%, 94.82%, and 107.64%, respectively, while the corresponding values with the minirhizotron method were 68.91%, 61.69%, 28.24%, 77.19%, respectively, in 2014; in 2015, these values were 90.37%, 82.47%, 61.11%, and 138.06%, respectively, for the soil coring method and 57.47%, 107.57%, 93.88%, and 78.66%, respectively, for the minirhizotron method.

### Cotton root length density at different soil depths under different nitrogen treatments as measured by the soil coring method and minirhizotron method

**Fig 3:**
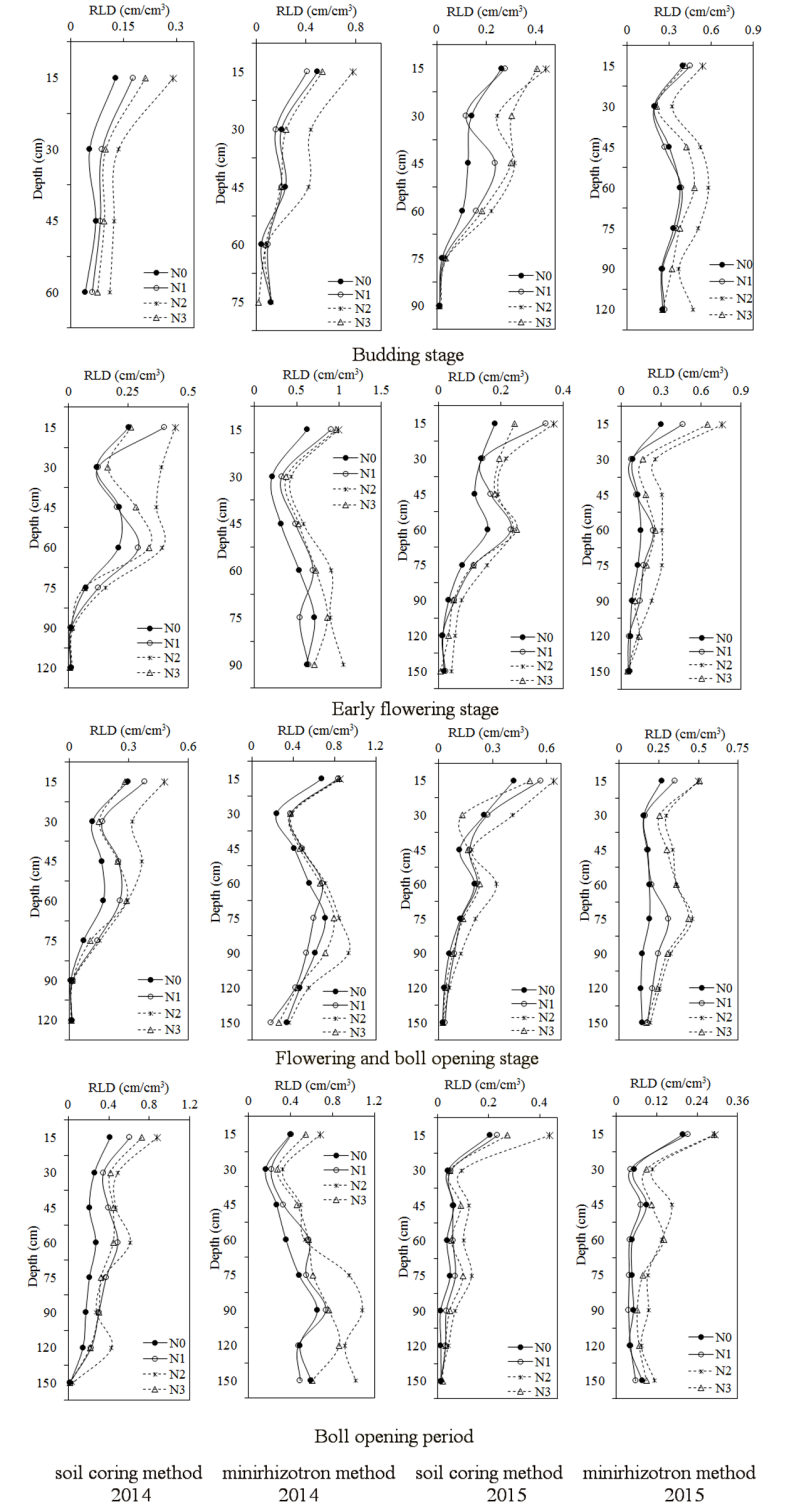
Changes in root length density at different soil depths under different nitrogen treatments using the soil core method and minirhizotron method.

As Fig 3 shows, compared with the soil coring method, the minirhizotron method resulted in higher root length density values at different soil layers, and the differences among treatments were more obvious. Furthermore, nitrogen effects on root length density at different soil depths were consistent between the two methods. The root length density at most soil depths first increased but then decreased as the fertilizer rate increased, especially in the shallow soil depths. Compared with N0, moderate nitrogen fertilization resulted in the greatest root length density, which markedly increased.

### Effects of nitrogen rate on the root distribution in different soil layers with the soil coring method and minirhizotron method at the flowering and boll opening period

**Fig 4:**
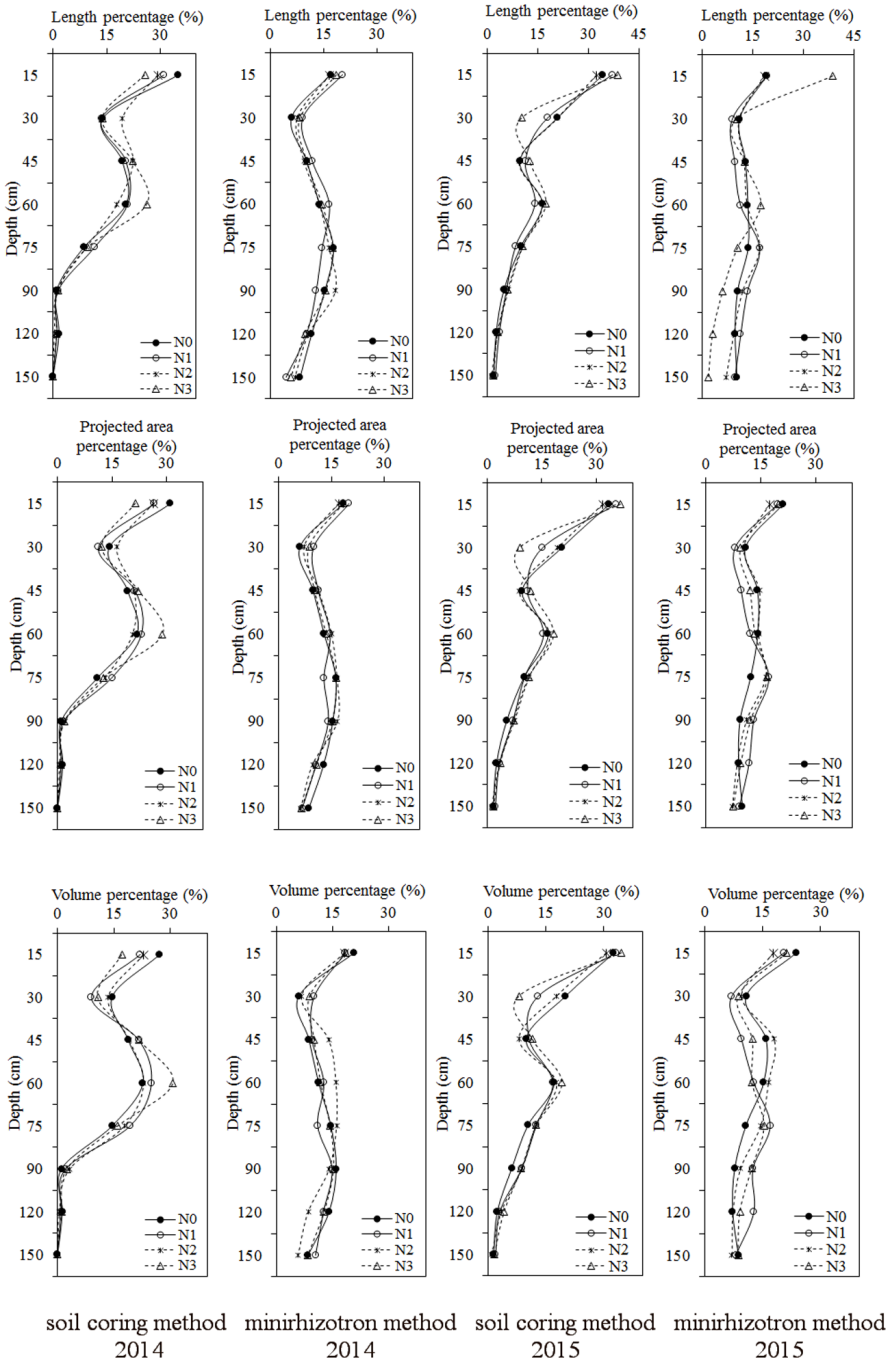
Percent change in root length, root projected area and root volume at different soil depths under different nitrogen treatments using the soil coring method and minirhizotron method.

The results showed that the root distribution in the shallow soil was greater than that in the deeper soil for both methods (Fig 4). The distribution in the shallow soil layers observed using the soil coring method was greater than the distribution values observed from the minirhizotron method. As recorded from the soil coring method, the main root indexes distributed in the 0–60 cm soil layer, including the root length, root projected area, and root volume, accounted for more than 85.87%, 82.27% and 77.67%, respectively, in 2014 and 78.73%, 76.12% and 73.11%, respectively, in 2015; the percentages observed using the minirhizotron method were greater than 47.48%, 47.07% and 46.77%, respectively, in 2014 and 48.67%, 48.96% and 49.76%, respectively, in 2015. In addition, the root distribution in the deeper soil layers was greater when measured using the minirhizotron method.

### Correlations between root morphology distributions at different soil depths measured using the soil coring method and minirhizotron method at the flowering and boll stage

The correlations between the root morphology distributions generally reached a significant level when measured by both methods (Table 2). The correlation coefficients between the root length density distribution, root projected area density distribution, and root volume density distribution and other indexes were greater than 0.346, 0.325, and 0.300, respectively, using the soil coring method and 0.383, 0.402, and 0.300, respectively, using the minirhizotron method in 2014; in 2015, the values were 0.484, 0.526, and 0.555, respectively, using the soil coring method and 0.628, 0.672, and 0.586, respectively, using the minirhizotron method. The correlation coefficients measured using the soil coring method were lower than those measured using the minirhizotron method. In addition, the relationships between root volume density distribution using the minirhizotron method and the other indexes were generally not significant.

**Table 2:** Correlations between root morphology indexes at the flowering and boll stage using the soil coring method and minirhizotron method.

Correlation coefficient	2014A-RLD	2014B-RLD	2014A-RPAD	2014B-RPAD	2014A-RVD	2014B-RVD	2015A-RLD	2015B-RLD	2015A-RPAD	2015B-RPAD	2015A-RVD	2015B-RVD
2014A-RLD	1											
2014B-RLD	0.383*	1										
2014A-RPAD	0.981**	0.418*	1									
2014B-RPAD	0.402*	0.971**	0.416*	1								
2014A-RVD	0.913**	0.455**	0.975**	0.430*	1							
2014B-RVD	0.346	0.804**	0.325	0.918**	0.300	1						
2015A-RLD	0.859**	0.484**	0.790**	0.523**	0.683**	0.496**	1					
2015B-RLD	0.628**	0.757**	0.633**	0.792**	0.630**	0.736**	0.727**	1				
2015A-RPAD	0.861**	0.533**	0.805**	0.566**	0.712**	0.526**	0.996**	0.763**	1			
2015B-RPAD	0.680**	0.706**	0.684**	0.749**	0.672**	0.720**	0.723**	0.975**	0.758**	1		
2015A-RVD	0.854**	0.585**	0.812**	0.610**	0.736**	0.555**	0.982**	0.797**	0.955**	0.791**	1	
2015B-RVD	0.730**	0.586**	0.722**	0.647**	0.689**	0.664**	0.721**	0.885**	0.749**	0.963**	0.773**	1

A: soil coring method; B: minirhizotron method; RLD: root length density; RPAD: root projected area; RVD: root volume density

* Significant at P≤0.05;

** Significant at P≤0.01.

### Correlations between root morphology densities at shallow and deep soil depths measured using the soil coring method and minirhizotron method

With respect to the 239 groups of root morphology density data within shallow soil depth (0–60 cm) and deep soil depth (60–150 cm), correlations between the two methods were analyzed (Table 3). The correlative coefficients of root length density, root projected area density, root surface area density, root average diameter density and root volume density of the two models were 0.687, 0.544, 0.508, 0.334 and 0.418, respectively, in shallow soil depth and 0.348, 0.277, 0.260, 0.143 and 0.190, respectively, in deep soil depth. The root morphology densities in shallow soil depth obtained by the two methods were significantly correlated, and the values were higher than the deep depth.

**Table 3:** Correlations between root morphology densities at shallow soil depth and deep soil depth measured using the soil coring method and minirhizotron method.

Correlation coefficient	Root length density	Root projected area density	Root surface area density	Root average diameter density	Root volume density
within shallow soil depth	0.687**	0.544**	0.508**	0.334**	0.418**
within deep soil depth	0.348**	0.277**	0.260**	0.143	0.190*

* Significant at P≤0.05;

** Significant at P≤0.01.

## Discussion

The main advantage of the minirhizotron method is continuous and in situ observation with high efficiency; thus, the minirhizotron method could reflect actual growth more accurately [33]. In order to further calibrate the minirhizotron method against traditional method, we studied the cotton root morphology and distribution under different nitrogen fertilization rates using the soil coring method and minirhizotron method. The response of most root traits to nitrogen supply showed similar trends for both methods. However, the results showed several interesting aspects, as described below.

### Comparison of the root sampling processes involving the soil coring method and minirhizotron method

On the one hand, the minirhizotron method has great advantages. The process of cotton root measurement was simpler with the minirhizotron method, and less time and labor were needed [34]. Because there are more involved processes and labor needed for the soil coring method, the error will be larger. In addition, many fine roots could be lost in the process of washing the soil block [35].

However, the sampling volume of the minirhizotron method was much smaller than that of the soil coring method, and the installation environment of the minirhizotron tubes and the soil texture can easily affect the results [36, 37], necessitating more replication. Although image capturing is fast, the analysis of the images is time consuming. The use of software to automatically analyze minirhizotron images may be able to solve this problem in the future [38].

### Comparison of root indexes under different nitrogen fertilization rates as measured by the soil coring method and minirhizotron method

The total root length density and the majority of the root length density in different soil layers, especially in the upper soil layers, first increased but decreased as nitrogen fertilization increased, and the same trend was observed for both methods. This finding indicated that a lack of nitrogen could restrain root growth and that excessive nitrogen did not increase root growth. The root length density measured via the minirhizotron method was higher than that measured via the soil coring method. This difference, by virtue of the loss of fine roots during the process of the soil coring method, was irreconcilable. However, this finding is different from Machado’s results [39], which showed that a black cover used to prevent light caused a decrease of the root length at the uppermost soil layers. In our study, we used covered white tubes outside the black cover, and the minirhizotron tubes were shaded by the cotton plants. And the tubes were installed the year before experiment.

Most roots are distributed within the shallow soil layers, as observed via both methods. Similar results have been reported [12, 40, 41]. However, the distribution in shallow soil layers was greater in the soil coring method than in the minirhizotron method. The percentages of roots distributed in the 0–60 cm soil layer observed using the soil coring method and minirhizotron method were more than 73% and 46%, respectively. This difference possibly occurred because the roots in the deeper soil layers were small and tender and could have been easily lost in the soil coring method. Moderate nitrogen fertilizer increased the percentage in the deeper soil layers. The results showed that proper nitrogen may promote root growth in deeper soil, which will not only accelerate root growth but also increase the use of nutrients in deeper soil.

The correlation coefficients between the root morphological indexes obtained using the minirhizotron method were higher than those obtained using the soil coring method, which indicated that the root morphology observed using the minirhizotron method was more accurate, to some extent. However, with respect to root volume, the minirhizotron method was less accurate. This difference could have been occurred because the roots were partially covered by the soil in the minirhizotron method, and the sampling volume was smaller. The correlations of the root morphology indexes between the two methods were generally significant in the shallow soil depth, which is consistent with the results of Jose’s research [42]. However, additional studies in which models are established to calibrate the root indexes are needed to understand the exact relationships between the two methods.

## Conclusion

The results in the present study have shown that the minirhizotron method may be suitable for the continuous monitoring of cotton roots under field conditions. Most of the correlations of the cotton root length and root distribution indexes were significant when measured by minirhizotron method and the soil coring method. A moderate amount of nitrogen increased the root growth, especially within the upper soil layers, which were observed by the two methods. In addition, the differences between the different nitrogen treatments were clearer and more accurate with the minirhizotron method than with the soil coring method.

## Supporting information

S1 Data Origin data for Figs 1, 2, 3 and 4. (XLSX)
